# Digital Tip Amputations from the Perspective of the Nail

**DOI:** 10.1155/2016/1967192

**Published:** 2016-11-13

**Authors:** Lloyd Champagne, Joshua W. Hustedt, Robert Walker, John Wiebelhaus, N. Ake Nystrom

**Affiliations:** ^1^Department of Orthopedics, University of Arizona College of Medicine Phoenix, Phoenix, AZ, USA; ^2^Arizona Center for Hand Surgery, Phoenix, AZ, USA; ^3^Department of Hand and Plastic Surgery, Stavanger University Hospital, Stavanger, Norway

## Abstract

The management strategy proposed herein for fingertip amputations advocates secondary healing with preservation of appearance as well as function. Conservative healing is more likely to result in a sensate, nontender, and cosmetically acceptable fingertip compared to surgical management in many clinical scenarios. This manuscript examines in detail the extent of fingertip injury and defines the relationship of injury to final fingertip outcome. A classification is presented, which allows adequate initial counseling regarding prognosis, and predicts the need for secondary corrective surgery.

## 1. Introduction

A reasonable treatment strategy for a fingertip amputation should consider both cosmetic and functional outcome. The conservative approach requires no surgical skill, has a low risk for complications, and is likely to result in a sensate, nontender, and cosmetically appealing finger [1–11]. Based on previous experience and literature evidence, many fingertip amputations can heal by secondary intention. The classification system that is adopted herein predicts the need for secondary corrective surgery.

## 2. Management

Any fingertip amputation through or distal to the germinal matrix should be considered for nonsurgical management. The exceptions are acutely oblique amputations or those with obvious gross damage to the germinal matrix. Healing by secondary intention is an excellent primary choice if the extent of damage to the germinal matrix and the potential for a functional nail are unclear.

**Figure 1:**
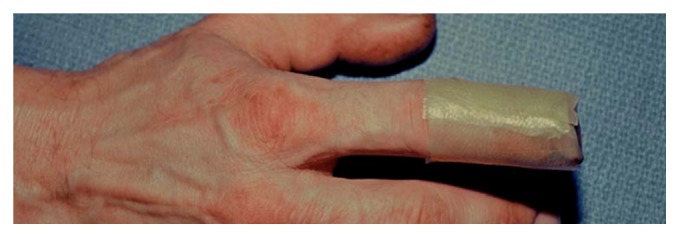
Simple covering for distal amputation injury.

Management of injuries suitable for secondary healing includes counseling the patient about options of wound care and providing information on ultimate function, including appearance of the injured finger. A digital block is sometimes provided for acute pain relief, and the wound is dressed. In order to minimize nail deformity, bone should seldom be shortened, even if it protrudes slightly beyond the level of amputation. It is not necessary to cover the exposed end of the distal phalanx with soft tissue. Any nonadhesive dressing material is likely to be adequate [1, 10–12], and wound care is simple, with soap-and-water cleansing and dressing changes once or twice weekly (Figure 1).

**Figure 2:**
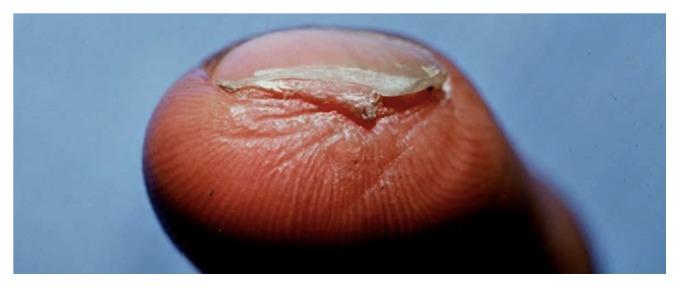


The initial tenderness subsides markedly at 7–10 days, and comfort rather than healing defines when patients are ready to return to work. Complete healing takes place within 4–6 weeks [3, 6, 9, 10, 12–19]; larger wounds with bone exposure [1, 8] require the longest time to heal. During the initial 1-2 weeks, the amputation stump undergoes little change except for the establishment of a granulation pad, which gradually covers the exposed bone. Thereafter, the wound contracts and expands around the surrounding skin and nail bed. The resulting scar is typically nontender, transverse in shape, and positioned under the nail (Figure 2).

## 3. Classification

**Figure 3:**
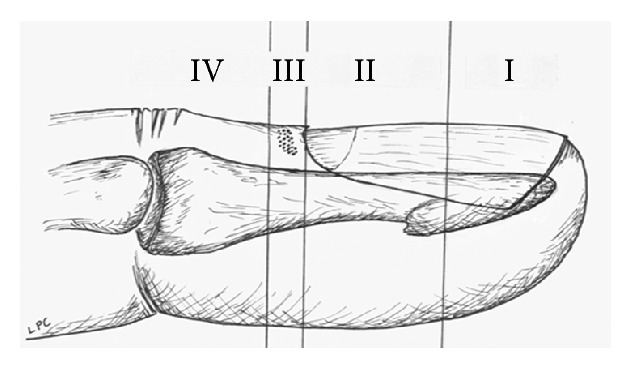


**Figure 4:**
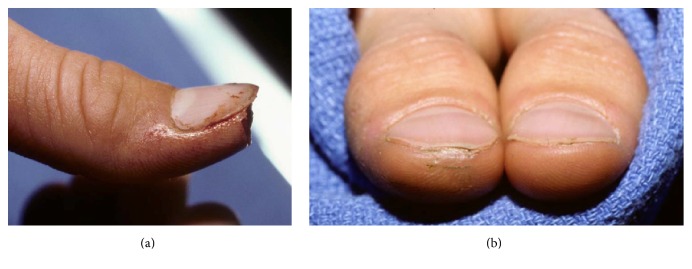


The following classification system is helpful in evaluating distal amputation injuries (Table 1 and Figure 3).* Type* I amputations include soft tissue only or soft tissue and bone, but with preservation of at least one-half of the nail bed (Figures 4(a) and 4(b)). Most of these fingers, particularly in children, heal with little or no evidence of the initial injury. When nail deformities (hook nail) occur, they are rarely severe [20]. Type I injuries are extremely unlikely to require secondary surgical correction.

**Figure 5:**
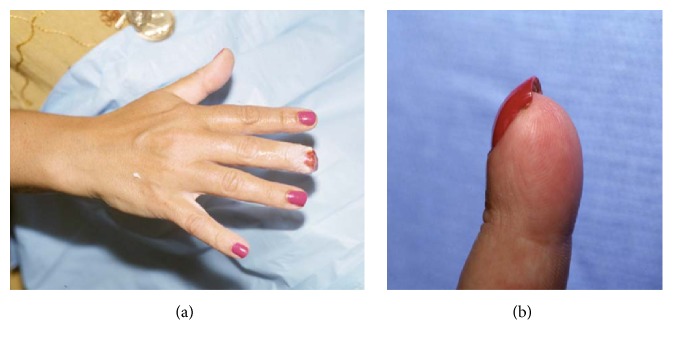


*Type II* amputations occur through the proximal half of the nail, distal to the cuticle. In these injuries, the regrowing nail has insufficient bony support and will develop a hook deformity. During the healing process, the nail bed expands to provide a shortened but potentially functional nail [11]. Should the patient request correction, an “antenna procedure” [21] or other revision for correction of the anticipated hook nail deformity is best performed 6–12 months after the initial injury (Figures 5(a) and 5(b)).

*Type III* amputations are proximal to the eponychial fold but distal to the flexor and extensor insertions. Since it is difficult to discern the extent of injury to the germinal matrix, we avoid primary ablation. This represents a risk for symptomatic nail remnants but also preserves the possibility of a functional nail. If the nail matrix survives intact, a nail will grow, but with a severe hook deformity. All Type III injuries are likely to require later surgery, either ablation of nail remnants or correction of a hook nail.

**Figure 6:**
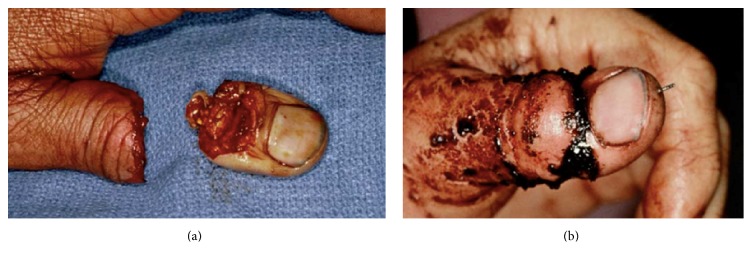


*Type IV* amputations have no potential of a nail apparatus unless a replantation is performed (Figures 6(a) and 6(b)). If replantation is not chosen, preservation of length is frequently no longer crucial, and secondary healing offers no advantage over primary closure or coverage. In some cases such as the thumb with significant soft tissue injury, if it is felt that primary closure would result in significant shortening, then flap coverage may be indicated. Additionally, there may be some significant benefit to preserving intact flexor and extensor insertions as well as the most distal interphalangeal joint.

**Figure 7:**
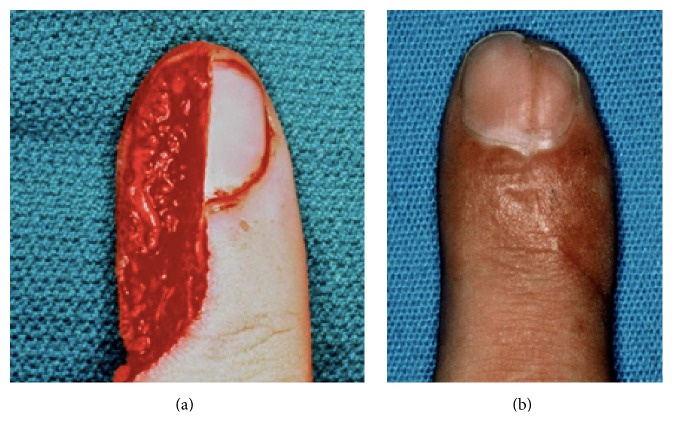


*Type V* injuries are oblique amputations with an angle less than 45° to the long axis of the finger (Figures 7(a) and 7(b)). These injuries typically require surgery, either replantation or flap coverage.

## 4. Discussion

The most frequent traumatic amputations to the upper extremity are distal to the distal interphalangeal joint [5, 22]. These seemingly mundane injuries represent a significant cost to society and to the affected individuals for treatment and from losses due to decreased production. Persistent cold intolerance [1, 8, 23–25], tenderness, and disfigurement [1, 8, 11] may have a major long-term impact on personal and professional activities [26]. In each case, the final outcome depends to some degree on the initial treatment, which may cause more disability than the injury itself. The treatment should therefore both optimize final appearance and function of the finger and minimize the risk for iatrogenic damage.

Conservative treatment requires no surgical skill or training and brings no risk for iatrogenic damage. This factor is important, since most cases are treated by physicians with no in-depth training in hand surgery [27]. The primary care physician can and should manage many fingertip amputations, whereas secondary nail reconstruction, if indicated, should be performed by a hand surgeon at a later stage.

*Primary direct closure* is unsatisfactory in most amputations of Types I–III. This type of repair requires either bone shortening [22, 28, 29] or suture under tension [22], both of which are likely to cause further deformity and other symptoms [22]. Trimming the bony support of the nail bed shortens the finger and increases the hook deformity. Primary closure may prevent the nail bed distraction that occurs when fingertips heal by secondary intention, and repair under tension increases the risk for cold intolerance and pulp tenderness [5, 9, 11, 19, 22, 28, 30].

*Free composite grafting* of the amputated tip has been reported as a successful technique [3, 14, 31–33]. We have not managed to duplicate these results, and reattached tissues have in our patients merely acted as temporary, biological dressings. In children in particular we prefer avoiding a surgical procedure [16].* Skin grafts* are associated with more tenderness, cold intolerance, and diminished sensitivity than what is seen after secondary healing [5, 11, 22, 28, 34, 35].

*Local flaps* [9, 18, 20, 23, 26, 31, 36–40] are tedious to perform and are associated with risk of flap failure [20, 30, 37, 40–43]. Furthermore, iatrogenic sensory loss is common even when experienced hand surgeons perform the surgery [22, 26, 36, 44]. The palmar advancement flap, originally described by Mennen and Wiese [45], is prone to cause interphalangeal flexion contracture [26] and, in the four ulnar digits, devascularization of the dorsal skin [43]. Additionally, with any of these flaps, bone shortening is sometimes required to complete closure [20, 21, 38, 42].

*Distant flaps* may be necessary for coverage of exceptionally large soft tissue defects but offer no advantage to secondary healing in Type I–III amputations. Like local skin flaps, they are associated with a certain rate of failure and other complications such as infection, joint stiffness, hyperesthesia, or poor sensation [18, 35, 37, 38, 44, 46–55].

*Microvascular reconstruction* including replantation or toe-pulp transfer, when successful, can provide excellent results and possibly reduce the risk of cold intolerance and painful neuroma [9, 33, 39, 49, 49, 56–59, 59–61]. The option is available to extremely few patients, since most cases are treated where microvascular skill is not immediately available.

*Healing by secondary intention* remains a preferred treatment because it provides the best possible functional and cosmetic result, with minimal risk of iatrogenic complications. In spite of its simplicity, the method requires adherence to a few basic principles. It is important not to remove bony support since this increases hooking of the regrowing nail [5, 17, 30, 38, 42, 62]. Sharp bone spicules can be trimmed, but a 1–3 mm protrusion of the phalanx rarely causes problems [1, 16].

The fear of bone infection has prompted surgeons to provide immediate soft tissue coverage [20, 30, 34]. Along with primary soft tissue coverage, antibiotics are commonly recommended in fingertip amputations where bone is exposed [3]. However, infections are rare [1, 3–6, 10–13, 16, 17, 28, 30, 60] when bone is left exposed in the fingertip for secondary healing, and prophylactic antibiotics are never used in our protocol. In contrast, infections can and do occur if the treatment includes primary coverage of the wound [22, 38]. An additional reason not to cover these wounds through primary surgery is that stable coverage interferes with wound contraction [3, 10]. This fact is frequently overlooked, and when secondary procedures are necessary it may be questioned, “Why not simply cover the wound at the time of injury?” The obvious reason is that the “biologic tissue expansion” and distal advancement of tissues that is caused by wound contraction are desirable. During this process, the nailbed expands distally and digital glabrous skin contracts to cover the denuded bone [3, 11, 13]. The primary application of flaps is hence detrimental to both the ultimate length of the nail and sensation of the finger. No primary procedure, except possibly replantation, has conclusively been shown to prevent nail deformities [19, 30, 31, 38, 41, 46, 59, 60, 62].

Healing times may be longer when tip amputations heal by secondary intention rather than after primary coverage [34], but the factors that define ability to work relate more to local tenderness than to the presence of a granulating wound. There is no evidence that granulating fingertips hurt more than fingertips that have been primarily covered. There is also no evidence that conservative treatment results in longer short-term disability than any other method of reconstruction. In fact, the time off work appears to average 3-4 weeks whether conservative management, skin grafting, primary closure, or flap coverage has been performed [1, 3–8, 10–13, 15, 17, 19, 28, 30, 35, 46].

Although healing by secondary intention remains our preferred treatment when indicated, there are scenarios where surgical management is performed. These include patients who cannot tolerate an open wound or patients that choose surgery after the informed consent discussion. Some patients simply do not like the idea of an open wound or think that for various reasons that surgery must be superior.

Several classification schemes for fingertip amputations have been presented to determine the treatment strategy [4, 8, 17, 57, 58, 61, 63]. Since we promote conservative treatment for most injuries, the classification that we propose serves mainly to assess the functional and cosmetic prognosis. With Type I amputations, the likelihood of a functional tip with minimal residual deformity is high. Type II injuries usually heal with some degree of hook deformity, requiring secondary surgery only if severe. Type III lesions result in a severely deformed nail, which may or may not be salvageable with corrective surgery. The prognosis is difficult to assess at the initial evaluation, but primary ablation of the germinal nail matrix certainly eliminates any chance of later restoration of a functional nail.

Type IV and V amputations typically require primary surgery [40, 61] and rarely require secondary procedures [33]. Secondary surgical procedures are with few exceptions limited to resection of nail remnants in Type III lesions or correction of hook nail deformities in Type II-III lesions [22, 26, 62]. The antenna procedure is our preferred approach to the hook nail [21].

## 5. Conclusion

There are a myriad of ways to manage fingertip injuries. Healing by secondary intention remains a preferred treatment strategy. This strategy avoids primary surgery and its pain and complications, while providing an excellent functional and cosmetic outcome. Secondary healing should be a part of the management algorithm for all fingertip injuries.

## Figures and Tables

**Table 1:** Classification system for amputation injuries.

Type of injury	Amputation	Treatment	Cosmetic outcome	Secondary surgery
I	Distal 1/2 of nail	Conservative	Excellent	None
II	Proximal 1/2 of nail	Conservative	Hook nail	Likely
III	Through germinal matrix	Conservative	Hook nail *or *nail remnants *or* absence of nail	Very likely
IV	Proximal to germinal matrix	Replantation *or* primary closure	Excellent with replantation	Not likely
V	>45° oblique	Replantation *or *primary closure	Variable	Not likely
